# Clinical laboratory diagnostics in dentistry: Application of microbiological methods

**DOI:** 10.3389/froh.2022.983991

**Published:** 2022-09-08

**Authors:** Rolf Claesson, Anders Johansson, Georgios N. Belibasakis

**Affiliations:** ^1^Division of Oral Microbiology, Department of Odontology, Umeå University, Umeå, Sweden; ^2^Division of Oral Diseases, Department of Dental Medicine, Karolinska Institutet, Huddinge, Sweden

**Keywords:** oral microbiology, clinical microbiology laboratory, periodontal diseases, endodontic infections, odontogenic abscesses, antibiotics, antimicrobials, dental treatment

## Abstract

Diagnosis and treatment in dentistry are based on clinical examination of the patients. Given that the major oral diseases are of microbial biofilm etiology, it can be expected that performing microbiological analysis on samples collected from the patient could deliver supportive evidence to facilitate the decision-making process by the clinician. Applicable microbiological methods range from microscopy, to culture, to molecular techniques, which can be performed easily within dedicated laboratories proximal to the clinics, such as ones in academic dental institutions. Periodontal and endodontic infections, along with odontogenic abscesses, have been identified as conditions in which applied clinical microbiology may be beneficial for the patient. Administration of antimicrobial agents, backed by microbiological analysis, can yield more predictable treatment outcomes in refractory or early-occurring forms of periodontitis. Confirming a sterile root canal using a culture-negative sample during endodontic treatment may ensure the longevity of its outcome and prevent secondary infections. Susceptibility testing of samples obtained from odontogenic abscesses may facilitate the selection of the appropriate antimicrobial treatment to prevent further spread of the infection.

## Background

The oral cavity is colonized by a high number of bacteria, consisting of more than 700 species. The consortium of microorganisms residing in our oral cavity is collectively identified as “oral microbiota,” yet often inadvertently used in the definition of “oral microbiome,” which refers to the summation of genes that constitute the oral microbiome [1]. Regardless of the definition, the oral microbiota and genes thereof are essential for regulating our immune system as a double-edged sword. While they can prime a beneficial immune response or establish a residential competition against exogenous pathogens, regional deregulation of the interrelationship between the microbiota and the host response (“dysbiosis”) will inevitably lead to oral diseases, such as gingivitis, periodontitis, dental caries, root canal infections, or oral candidiasis. Identifying such changes in the oral microbial ecology necessitates clinical laboratory diagnostics, which are implemented by evaluating the microbiota known to be associated with the type of disease under investigation. It is important to distinguish between microbial analytics for experimental research purposes, which are carried out for etiological discovery and concept validation purposes, from clinical laboratory analytics, which are done on a patient-to-patient (or sample-to-sample) basis to support the diagnostic and treatment outcome [2, 3]. In a nutshell, the primary purposes of performing clinical oral microbiological sample analysis in oral healthcare are (a) to conduct an early risk assessment for developing oral diseases, (b) to assist in the diagnosis of oral diseases, (c) to retrieve auxiliary information for supporting the treatment planning, and (d) to follow up treatment outcome. This study will focus on clinical laboratory microbiological analytics, primarily for periodontal diseases, endodontic infections, and odontogenic abscesses, attempting to highlight currently meaningful practicalities for dentistry.

## Periodontal diseases—open diagnostic questions

Periodontal disease, or periodontitis, is initiated by the biofilm forming on the tooth surface that triggers an inflammatory immune response by the surrounding periodontal tissue [4]. Hence, periodontitis is of microbial etiology; thus, identifying microbiological changes in the periodontal niche could be of diagnostic value. The routine laboratory microbial diagnostic methods currently available do not facilitate the open-ended screening of the oral microbiome. This is at present achievable only for research purposes, necessitating expensive and tedious analytical platforms. While research is gradually paving the way for whole microbiome chairside analyses [5], we hereby aim to summarize and refresh more “classical” approaches that could bridge the current gap between clinical and laboratory diagnostics for periodontal diseases.

**Figure 1:**
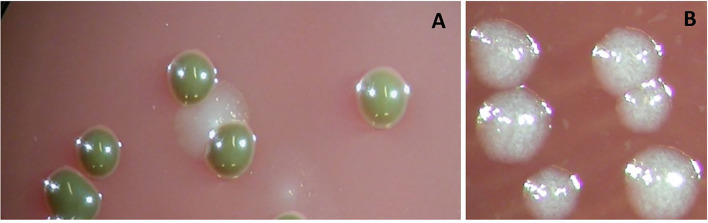
Colonies of *Porphyromonas gingivalis* grown anaerobically on Columbia-base blood agar are brownish and indol-negative **(A)**. Colonies of *Tannerella forsythia* grown anaerobically on Columbia-base blood agar are speckled and give a positive reaction to the N-acetyl muramic acid substrate **(B)**.

Fundamental laboratory analyses have been largely focusing on the identification of Socransky's “red complex” species, which includes *Porphyromonas gingivalis, Treponema denticola*, and *Tannerella forsythia* (Figure 1), or the somehow less pathogenic “orange complex” species, consisting among others of *Fusobacterium nucleatum* and *Prevotella* spp. Considerable focus on cultivation-based microbiology has also been placed on the identification of *Aggregatibacter actinomycetemcomitans*, a species associated with periodontitis occurring in adolescents or young adults, or *Filifactor alocis*, which is a more recently identified organism. In addition, for species identification, it can be beneficial to use biochemical or molecular methods to identify or even measure the expression of species-specific virulence factors, such as the leukotoxin of *A. actinomycetemcomitans* [6] or the cysteine proteinases (gingipains) of *P. gingivalis* [7]. Measurement of the expression of virulent factors may reveal the degree of virulence to the clinical isolate of the given species. This is discussed in further depth in the bacterial genotypic section below. *Filifactor alocis* (earlier *Fusobacterium alocis*) is, in contrast to most other periodontal pathogens, a gram-positive species, the identification of which has also attracted recent diagnostic attention for periodontitis [8].

While these species are clearly associated with the disease, it is hard to distinguish whether they are contributing to the causation of the disease or if their presence at an already diseased site results from favorable ecological conditions for their survival. Whatever the case, their clear association with the disease signifies an argument for investigating their presence at an affected periodontal site for diagnostic purposes.

## Available methods for quantifying periodontal pathogens in infected sites

This section provides an overview of methods commonly used in a clinical oral microbiology laboratory for quantifying bacterial species present in samples obtained from the dental clinic. The sampling technique is also a determinant parameter for this purpose, so we include it in our discussion.

**Figure 2:**
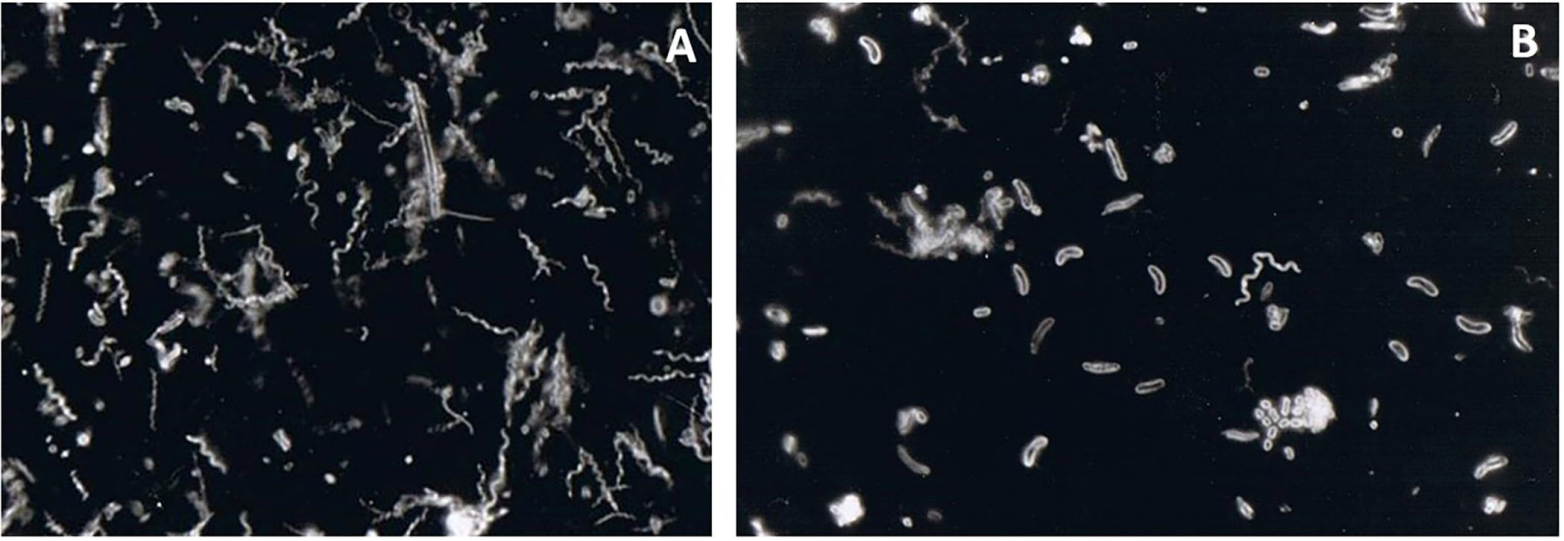
Darkfield microscopy observation of a subgingival biofilm sample obtained from a periodontal pocket. The sample was collected by paper points and transported to the laboratory in an anaerobic transport medium. It was then dispersed, and a small aliquot of the resulting suspension was placed on an objective glass and observed under dark field microscopy. High proportions of spirochaetes **(A)** and long curved motile rods **(B)** were observed. These are very characteristic bacterial “morphotypes” found in deep periodontal pockets. Spirochaetes are motile by nature. Long curved motile rods can represent primarily *Campylobacter* spp. and *Selenomonas* spp. within a periodontal pocket environment—magnification x1000.

**Figure 3:**
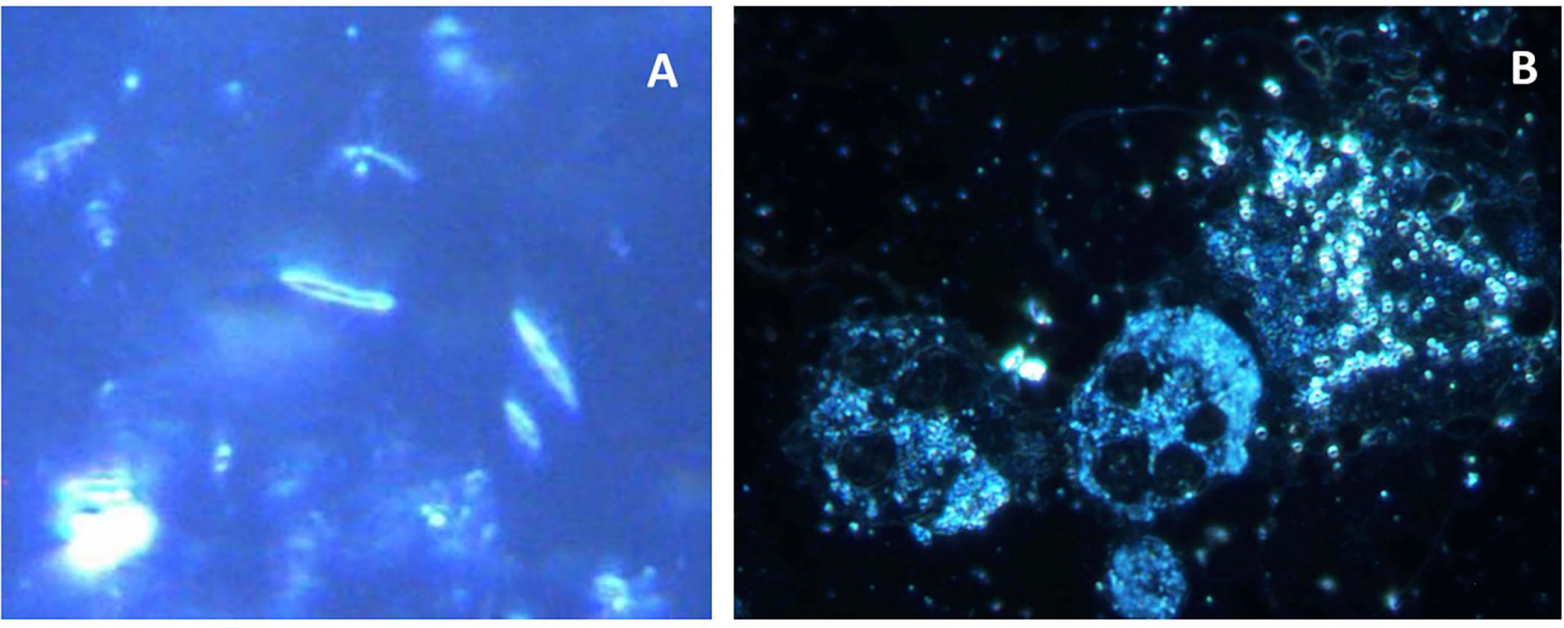
Darkfield microscopy of a sample obtained from a periodontal pocket. Elongated rod-shaped and fusiform bacteria are seen in the left figure. The neutrophils provide a front line of defense against the growing biofilm in an infected periodontal pocket. Thus, they can often be detected by microscopy within periodontal pocket samples. In the figure, elongated rod and fusiform bacteria can be identified **(A)**. Both activated neutrophils and non-activated neutrophils (displaying higher cytoplasmic granulation) can also be identified **(B)**. A high number of coccoid bacteria is seen close to the activated neutrophil. An increased number of neutrophils indicates rapid ongoing tissue damage—magnification x1000.

**Figure 4:**
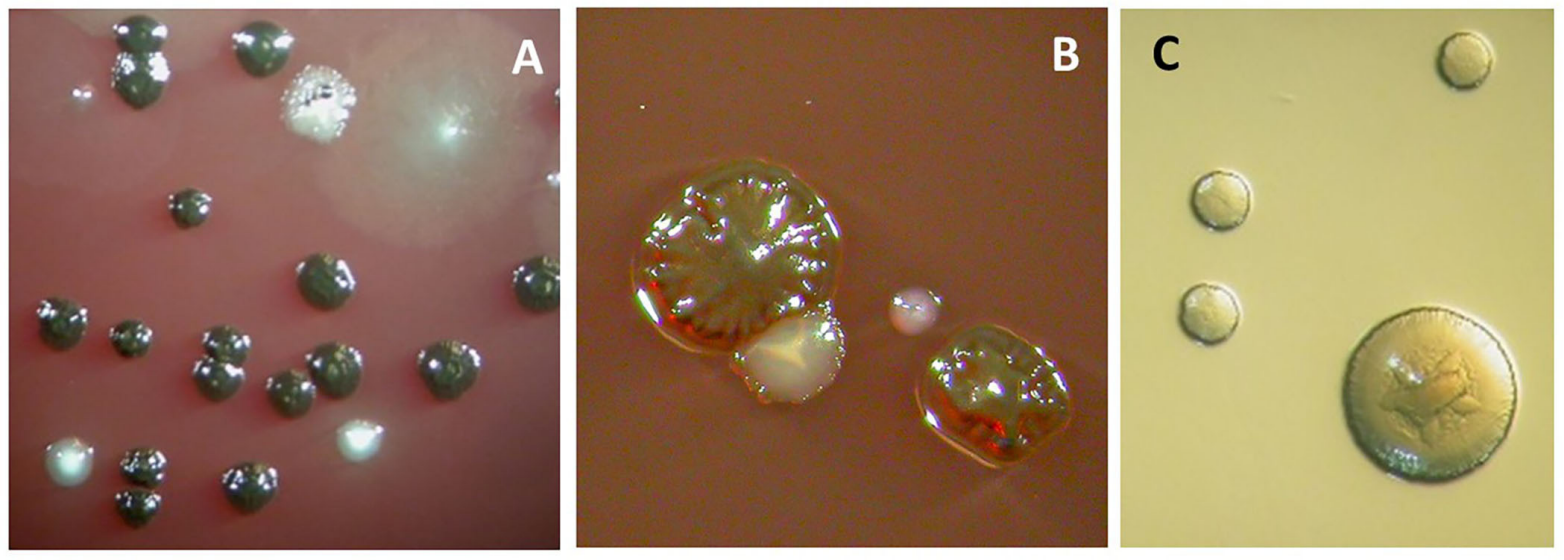
Anaerobic cell culture analysis of a subgingival biofilm sample obtained from a periodontal pocket. The sample was collected by paper points and transported to the laboratory in an anaerobic transport medium. It was then dispersed by “vortexing” and diluted up to 10,000 times before being spread on Columbia-base agar plates containing blood, hemin, and vitamin K and on an *Aggregatibacter actinomycetemcomitans*-specific medium (TBV) (serum was omitted, [9]). The blood agar plates were incubated anaerobically for 1 week, and the resulting colony-forming units (representing viable bacteria) were counted. **(A)** The observed colonies represent *Prevotella intermedia/nigrescens* (black-pigmented) and *Parvimonas micra* (white ones toward the bottom). **(B)** The observed colonies represent *Porphyromonas gingivalis* (rough-type colony, black-pigmented), *Aggregatibacter actinomycetemcomitans* (white colony with enclosed stare shape tangible to the *P. gingivalis* colony), and *Tannerella forsythia* (small while single colony to the right). **(C)** *Aggregatibacter actinomycetemcomitans* colonies growing on TBV-medium, which were incubated for 3–5 days in an aerobic atmosphere containing 5% CO_2_.

Visual identification of the periodontal microbiota was performed by microscopy. For instance, dark-field microscopy of samples from infected periodontal pockets renders an illustrative picture of the condition of the pocket. High proportions of spiral and motile organisms, curved motile rods, and fusiforms are associated with heavily diseased periodontal pockets (Figure 2). Neutrophils are often detected among these samples, indicating that the immune response is activated (Figure 3). Originally, quantifying selected bacterial species from samples collected from periodontal pockets has been performed with cultivation-based methods (Figure 4) under various atmospheric conditions (Table 1). In later years, nucleic acid-based amplification or hybridization methods or high-throughput sequencing platforms were developed, which have substantially contributed to the expansion of the number of bacterial phyla, genera, and species identified in the oral cavity. One of the biggest debates about using cultivation-based methods over nucleic acid-based ones is that the latter do not distinguish between living and dead bacteria, for better or worse. While cultivation by default ensures the enumeration of only living bacteria, DNA-based methods detect both living and recently dead bacteria. Bacterial death could already occur at the site of infection due to immune defense activity or even during the transport of the sample to the laboratory. On the other hand, DNA-amplification methods do not require the presence of living bacterial cells and thus offer great sensitivity as they allow small DNA amounts to be amplified exponentially [10]. It should be acknowledged that there is no perfect quantification method; thus, the selection should depend on the question to be addressed (Table 2). For instance, if the efficiency of an antimicrobial is to be tested, viable bacterial cultures should be selected. In contrast, for verification of bacterial presence at very low quantities, a PCR-based quantification should be preferred.

**Table 1:** Atmospheric growth conditions for different bacteria.

Strictly aerobic	→	Approximately 20% oxygen
Capnophilic	→	Oxygen and 5–10% carbon dioxide
Microaerophilic	→	1–5% oxygen
Facultatively anaerobic	→	Presence or absence of oxygen
Strictly anaerobic	→	Absence of oxygen (low redox potential)

**Table 2:** Microbial detection methods.

**Cultivation methods**
**Advantages:**
Bacterial isolates can be better characterized
Can distinguish aerobic from anaerobic species
Detection of unexpected cultivable bacteria
Use of internal standards
**Disadvantages:**
Non-cultivable bacteria will be undetected and thus overlooked
Identification of bacterial isolates requires knowledge and experience
Only living bacteria can be detected for good and bad
Transport medium for keeping the bacteria alive is required
Time-consuming
**DNA-based methods**
**Advantages:**
Non-cultivable bacteria can be detected
Rapid handling of samples—early availability of analysis results to the clinician
**Disadvantages:**
Bacterial isolates cannot be collected
Separating living and dead bacteria is impossible
DNA content varies between bacteria and creates inconsistency in generating internal standards for absolute quantification

Due to their different advantages and disadvantages, choosing an optimal microbial diagnostic method is virtually impossible. For optimal quantitative determination of clinical samples, both cultivation and DNA-based methods are recommended. The interpretation of the results should be made with caution because, unlike medical mono-infections, the presence of a species does not necessarily imply the occurrence of the disease.

Both DNA-based and cultivation-based methods face similar challenges. In contrast, when using DNA-based methods for quantification, there is an additional requirement to normalize the target species against the total bacterial concentration in the sample. The quantification of the selected species is achieved by species-specific primers, whereas the total bacterial content is quantified by amplifying the pan-bacterial 16s rRNA gene. The challenge that arises here is that the number of copies differs substantially between different bacterial species, which may lead to erroneous conclusions on the total bacterial presence in the sample, as some species may be over-represented, while others are under-represented based on their inherent number of 16S rRNA copies per cell [11]. If bacterial cultivation is the method of choice for quantification, the total viable count (TVC) can be used to normalize the bacterial content of a given sample. The quantitative calculation of the TVC of different species in a sample should be accompanied by a measurement of the sample's size (volume or weight); sole sampling of periodontal pockets with paper points or curettes does not ensure this. Normalization of TVC against the sample size provides information on the absolute numbers of a species, as well as its relative proportions or density in the sample. Increased numbers and/or proportions would indicate that the clinician needs to deliver additional scaling and root planning rounds for the biofilm removal or active reduction.

Sampling processes should be pre-considered when aiming at the quantification of selected species. It is plausible that the sample size is greater when using a curette than a paper point. Curette sampling may also be more efficient in collecting early colonizing bacteria when scrapping the tooth surface, whereas paper points may more efficiently collect loosely attached (late colonizing) bacteria from the outskirts of the biofilm mass. There can indeed be variations in the quantification outcomes when using different sampling principles to identify selected members of the pocket microbiota. Such differences have been demonstrated in the case of *A. actinomycetemcomitans* when sampled by paper points vs. curette, where detection frequency varies between the two approaches. On the other hand, there seems to be consistency in the detection frequency and levels of the “red-complex” species irrespective of the sampling technique [12].

**Figure 5:**
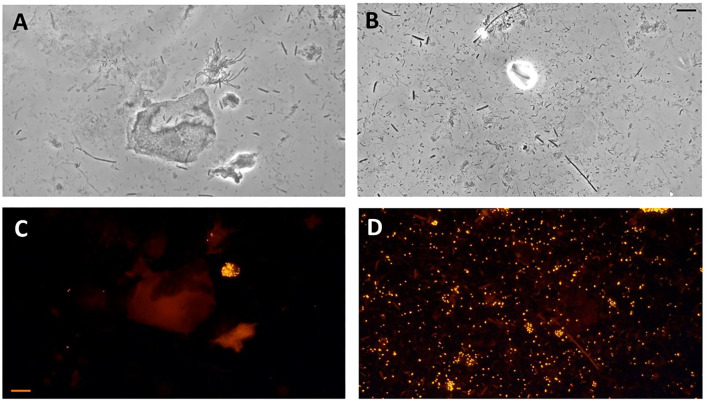
Photomicrographs of subgingival biofilm samples from the periodontitis pockets of two patients with periodontitis. Direct light microscopy **(A,B)** and fluorescence *in situ* hybridization (FISH) microscopy of the same samples using fluorescence-labeled 16S rRNA-oligonucleotide probes specific for *Aggregatibacter actinomycetemcomitans* **(C)** and *Porphyromonas gingivalis* **(D)** (Scale bars = 10 μm).

Developing species-specific probes for periodontal microbial diagnosis has enabled the microscopic identification of species in a collected subgingival plaque sample. They may entail both serological utilities (monoclonal or polyclonal antibodies against species-specific surface antigens) and single-stranded oligonucleotide sequences (probes) that are complementary to sequences of the genomic DNA or ribosomal RNA of a specific species. Coupled with epifluorescence microscopy, these principles have led to the development of immunofluorescence or fluorescent *in situ* hybridization (FISH) (Figure 5) methods that provide additional sensitive diagnostic tools for periodontal pathogen detection [13, 14].

## Bacterial genotyping for diagnostic support and risk classification

Genotyping is a suitable tool to distinguish microbial virulence [15], as intra-species genetic diversity can yield both harmless and virulent variants of a single species [16]. The most obvious example where genotyping contributes valuable information to clinicians is the subtyping of *A. actinomyctemcomitans* [17]. It was first shown that isolates from young individuals with severe periodontitis differed from those obtained from periodontally healthy individuals [18]. Molecular characterization of the isolates showed a high prevalence of serotype b with enhanced leukotoxicity in young periodontitis patients [19]. Later, the JP2-genotype was identified among the highly virulent serotype b isolates [20], which was found to be due to a partial deletion in the promoter region of the leukotoxin gene [21]. The JP2-genotype could be found frequently in oral samples from young individuals living in North- or West-Africa but occasionally in samples from young periodontitis patients of other origins [17, 21, 22]. The carriage of the *A. actinomycetemcomitans* JP2 genotype is a reliable risk marker for developing periodontitis in young individuals [23, 24]. More recently, a marker for serotype b isolates with enhanced leukotoxicity that includes both JP2 and non-JP2 genotypes have been detected, providing additional genomic variations [25, 26]. Tracing collectively these genotypic characteristics of *A. actinomycetemcomitans* may be important for determining the risk for periodontal disease in younger populations.

## The example of *A. actinomycetemcomitans* in periodontal microbial diagnostics

For diagnosis and treatment purposes, proportions of periodontitis-associated bacterial species in samples from periodontitis patients have been determined for many years. Increased proportions of pathogens in the periodontal pocket could act as risk indicators for the progression of periodontitis. As such, pocket detection of *A. actinomycetemcomitans* is considered an essential microbiological diagnostic utility for localized juvenile periodontitis, as it can colonize >90% of all affected sites and at high levels and proportions [27–29]. As an example carried out in our labs, in the case of a 33-year-old patient diagnosed with aggressive periodontitis, the cultivation of periodontal pocket biofilm (pooled samples from three pockets) indicated a total bacterial load of 1.1 million anaerobes, 78% of which proved to be *A. actinomycetemcomitans* (confirmed JP2 clone), but no *P. gingivalis, Porphyromonas intermedia*/*Porphyromonas nigrescens, T. forsythia, Campylobacter rectus*, or *Parvimonas micra* were detected. There was also a confirmed history of aggressive periodontitis in the patient's family. The recommended treatment, in this case, included adjunctive systemic administration of metronidazole and amoxicillin [30]. This combined antibiotic scheme results in the subgingival elimination of *A. actinomycetemcomitans* and a clinical improvement associated with its absence as high as 96.6% [31]. On the other hand, patients still positive for *A. actinomycetemcomitans* showed a significantly higher bleeding tendency after therapy [32]. Of interest, when determining the presence of *A. actinomycetemcomitans* isolates in a population of 1,445 younger and older (35 years' cut-off), the prevalence and proportions of plaque were higher among younger than older patients, who also displayed a higher prevalence of serotype b, irrespective of ethnicity. Hence, the age of the patient carrier may be a discriminating factor for the presence and genotype of *A. actinomycetemcomitans* in clinical biofilm samples. This information may assist in the more accurate diagnosis of the form of periodontitis [17].

The message here to the clinicians seeking auxiliary microbial diagnostics is to select a treatment strategy that results in the clinical restoration of periodontal health concomitantly with establishing a health-associated microbial ecology. Therefore, reduced detection or total elimination of such “surrogate marker” pathogens may reduce the risk of disease relapse and ensure long-term maintenance of successful treatment outcomes.

## Endodontic infections

Untreated caries may spread to the pulp tissue region, inadvertently leading to root canal infections. These are compositionally mixed non-specific infections of both gram-positive and gram-negative bacteria, as well as aerobic and anaerobic bacteria. Microorganisms of the initial pulp invasion cause primary endodontic infections during deep dental caries. In contrast, secondary endodontic infections are caused by microorganisms introduced into the root canal following treatment [33]. Different types of endodontic infections, including primary apical periodontitis, secondary apical periodontitis, and apical abscess, display diverse microbiota with differential abundances but with no clear microbial clustering according to diagnosis [34]. Microbiological differences between symptomatic and asymptomatic forms have yet to be delineated [35]. Persistent endodontic infections are caused by microorganisms that are part of either a primary or secondary infection that resisted chemo-mechanical debridement procedures and survived within the treated root canals. As the distinction between persistent and secondary infections remains clinically challenging, these conditions tend to be regrouped under the same pathological entity [33]. While primary infections are characterized by <50 gram-negative anaerobic species, persistent/secondary infections are dominated by up to 20 gram-positive facultative anaerobes, including *Streptococcus* spp., *Lactobacillus* spp., *Actinomyces* spp., and *Enterococcus* spp. The latter is a characteristic of persistent/secondary infections and an essential microbial diagnostic trait.

Maneuvering restrictions in the sampling process of infected root canals or their periapical region may hinder the topographic accuracy of the sampled site and, thus, the interpretation of the results [34, 36]. When endodontic therapy is warranted, the root canal must be disinfected of the contaminant microorganisms before being sealed off and restoring the tooth. To ensure that the root canal is sterile, it should ideally be sampled and analyzed by cultivation before filling. A sampling of the root canal can be performed by a sterile paper-point, whereas cultivation can take place on blood agar plates for up to 1 week under anaerobic conditions. Cultivation of samples obtained from a non-sterile root canal may yield a variety of microorganisms, some of which may prove to be resistant to dis-infective treatment. Examples of such bacteria are *Enterococcus* spp., *Actinomyces* spp., and *Propionibacterium* spp. It also displays an association with secondary endodontic infections [33]. Further identification of the bacterial colonies could be performed by biochemical methods, MALDI-TOF, or PCR—that is, sequencing of species-specific genes or species-specific parts of the 16S rRNA gene.

Overall, a sterile root canal can be safely sealed with a highly predictive treatment outcome, whereas confirmation of a persistently infected root canal would be a contraindication for sealing that necessitates further disinfection. With this in mind, the message to the clinician is that confirmation of the absence of viable bacteria in the sampled root canal may secure a successful endodontic treatment outcome. Therefore, the meaningful microbial diagnostic aspect here is to prevent secondary root canal infections.

## Odontogenic abscesses

**Figure 6:**
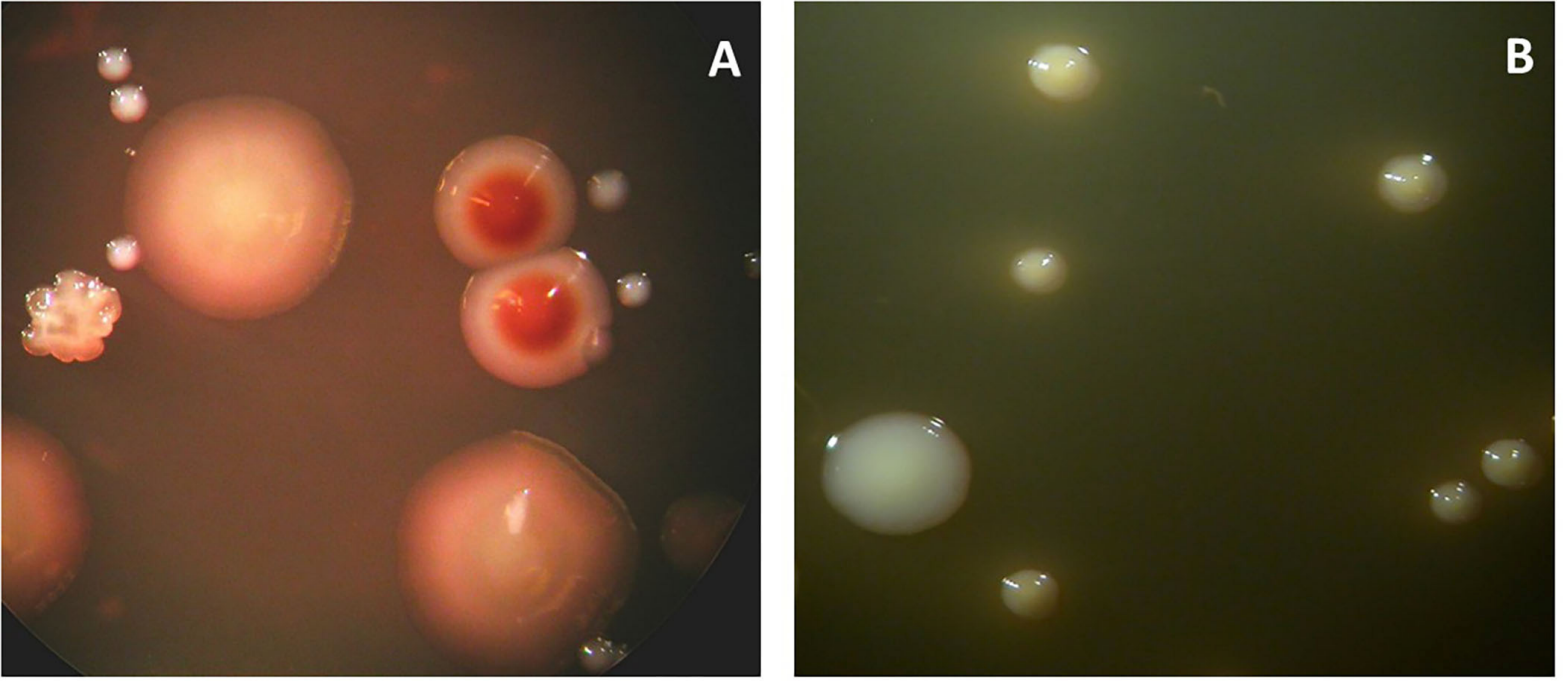
Microbial cultures of a sample obtained from the odontogenic abscess. The sample was transported in an anaerobic medium to the laboratory and incubated under anaerobic **(A)** or aerobic **(B)** conditions. The total viable counts were 12 × 10^7^ and 6.3 × 10^5^, respectively. The percentage of counts of specific colony types is provided below. **(A)** Identified colony types: *Actinomyces odontolyticus* (2%) (two brownish colonies). *Peptococcus stomatis* (4%) (three big colonies). *Parvimonas micra* (90%) (small white colonies). *Fusobacterium nucleatum* (1%) (rough colonies). **(B)** Identified colony types: *Enterococcus faecalis* (24%) (big colonies). *Streptococcus anginosus* (76%) (small colonies).

Progressive root canal infections may evolve toward extra-radicular odontogenic infections, which are purulent collections that infiltrate orofacial tissues. These conditions can exacerbate and devolve into potentially life-threatening infections and may therefore require antibiotic administration. They can eventually find their way through the alveolar bone to the soft gingival or vestibular mucosa and develop a purulent odontogenic abscess, which usually discharges its purulent content into the oral cavity via a duct. For diagnostic purposes, it is possible to sample the abscess duct with a syringe and process it in the microbiological laboratory for analysis. Samples must be incubated on cultivation plates for a prolonged period (e.g., at least a week) under both aerobic and anaerobic conditions. The microorganisms detected in the abscesses are frequently streptococci, *Actinomyces israelii*, or strictly anaerobic bacterial species such as *Parvimonas* spp. [34] (Figure 6). The isolated bacterial colonies are subsequently subjected to antimicrobial AST on different agents, as described in the next section.

## Antimicrobial sensitivity testing

Although oral diseases are mostly described as infections, the usage of antimicrobials to treat them is rather limited. This is partly due to the global increase in antibiotic resistance and their reduced efficiency in polymicrobial biofilms, which poses a challenge when choosing the appropriate antimicrobials. Today, amoxicillin, together with metronidazole, is used as an adjunctive option when treating refractory or aggressive forms of periodontitis [37]. In the case of aggressive periodontitis, the target organism has typically been *A. actinomycetemcomitans*. However, routine AST of *A. actinomycetemcomitans* clinical isolates to amoxicillin and metronidazole is rather dubious, as there are no species-specific breakpoints for these antimicrobials, nor have any amoxicillin-resistant strains been reported [38].

When treating root canal infections, antimicrobials are usually not used. Nevertheless, deep odontogenic infections that may develop into abscesses or spread across and beyond the soft oral tissues will necessitate the use of a suitable antibiotic. Hence, following cultivation of the sampled abscess, isolated bacterial colonies are subjected to AST on different agents. For this test, several other methods, such as agar dilution, broth dilution, and disc diffusion assays, are available [39]. The antibiotic choice is based on the AST results and may include benzyl-penicillin, amoxicillin, erythromycin, or metronidazole. However, as odontogenic abscesses are often polymicrobial infections, choosing a single antibiotic for each patient's case could be challenging. Odontogenic abscesses are perhaps the only type of oral infection that requires AST for successful treatment due to their life-threatening ramifications if left untreated.

## Conclusion

Clinical oral microbiology is a particularly strong aid in the armament of the dental clinician, yet it is most often overlooked or disregarded. With this review, we wish to highlight that “conventional” clinical microbiology laboratories, often integrated within dental schools, could provide important diagnostic and treatment planning information to in-house and external clinicians alike.

We have identified three types of common oral diseases or pathologies where the routine diagnostic potential is readily applicable. These include (a) aggressive or refractory forms of periodontitis (e.g., unresponsive to treatment) to facilitate the diagnosis and support selection of specific antimicrobials, (b) root canal infections, confirming the absence of microorganisms and sterility of the root canal before sealing, and (c) odontogenic abscesses, for selecting a suitable antibiotic following AST.

Last but not least, future innovations may allow the performance of microbiological assays in a chair-side fashion within the dental clinic without the need to dispatch the samples to a dedicated microbiology laboratory. These molecular platforms enable a very rapid sampling-to-answer pipeline (i.e., within a patient session). Chair-side assays under development include molecular quantification of periodontal or cariogenic species [40–42] or the detection of antibiotic resistance genes [43] within oral samples.

## Author contributions

All authors have contributed to the conception, design, and drafting of the manuscript.

## Funding

This work was supported by Karolinska Institutet Strategic Funds and the KI/SLL Styrgruppen för Odontologisk Forskning (SOF) Dnr. 4-823/2019 (GB).

## Conflict of interest

The authors declare that the research was conducted in the absence of any commercial or financial relationships that could be construed as a potential conflict of interest.

## Publisher's note

All claims expressed in this article are solely those of the authors and do not necessarily represent those of their affiliated organizations, or those of the publisher, the editors and the reviewers. Any product that may be evaluated in this article, or claim that may be made by its manufacturer, is not guaranteed or endorsed by the publisher.
